# Massive Activation of GABA_A_ Receptors: Rundown, Ionic and Neurodegenerative Consequences

**DOI:** 10.3390/biom15071003

**Published:** 2025-07-13

**Authors:** Sergey A. Menzikov, Danila M. Zaichenko, Aleksey A. Moskovtsev, Sergey G. Morozov, Aslan A. Kubatiev

**Affiliations:** Institute of General Pathology and Pathophysiology, 8, Baltiyskaya st., Moscow 125315, Russia; danilamihailovich@mail.ru (D.M.Z.); bioinf@mail.ru (A.A.M.); biopharm@list.ru (S.G.M.);

**Keywords:** GABA_A_ receptors, chloride, bicarbonate/pH, transport mechanisms, ionic plasticity, seizure activity

## Abstract

The GABA_A_ receptors, through a short-term interaction with a mediator, induce hyperpolarization of the membrane potential (*V_m_*) via the passive influx of chloride ions (Cl^−^) into neurons. The massive (or intense) activation of the GABA_A_Rs by the agonist could potentially lead to depolarization/excitation of the *V_m_*. Although the ionic mechanisms of GABA_A_-mediated depolarization remain incompletely understood, a combination of the outward chloride current and the inward bicarbonate current and the resulting pH shift are the main reasons for this event. The GABA_A_ responses are determined by the ionic gradients—neuronal pH/bicarbonate homeostasis is maintained by carbonic anhydrase and electroneutral/electrogenic bicarbonate transporters and the chloride level is maintained by secondary active cation–chloride cotransporters. Massive activation can also induce the rundown effect of the receptor function. This rundown effect partly involves phosphorylation, Ca^2+^ and the processes of receptor desensitization. In addition, by various methods (including fluorescence and optical genetic methods), it has been shown that massive activation of GABA_A_Rs during pathophysiological activity is also associated with an increase in [Cl^−^]_i_ and a decline in the pH and ATP levels in neurons. Although the relationship between the neuronal changes induced by massive activation of GABAergic signaling and the risk of developing neurodegenerative disease has been extensively studied, the molecular determinants of this process remain somewhat mysterious. The aim of this review is to summarize the data on the relationship between the massive activation of inhibitory signaling and the ionic changes in neurons. The potential role of receptor dysfunction during massive activation and the resulting ionic and metabolic disruption in neurons during the manifestation of network/seizure activity will be considered.

## 1. Introduction

Pentameric ligand-gated ion channels (pLGICs) are classic mediators of electrochemical signaling in the central nervous system of most kingdoms of life [1,2]. The cys-loop receptors in this family directly transduce the binding of an agonist into the opening of an intrinsic ion channel pore, enabling the passive conduction of cations or anions. These processes induce decremental fluctuations in the transmembrane potential (*V_m_*) that dynamically control/modify the neuronal excitation/inhibition balance in the brain [3]. pLGICs have similar cylindrical structures, consisting of five subunits arranged around a central axis [4,5]. The general features of each subunit include a large hydrophilic extracellular domain (ED), four transmembrane domains (TMDs), where TMD2 forms the ionic pore, and a small extracellular C-terminal region [2]. pLGICs are the object of extensive research because of their role in regulating synaptic signaling in the healthy brain and their involvement in various neurological disorders [6].

**Figure 1:**
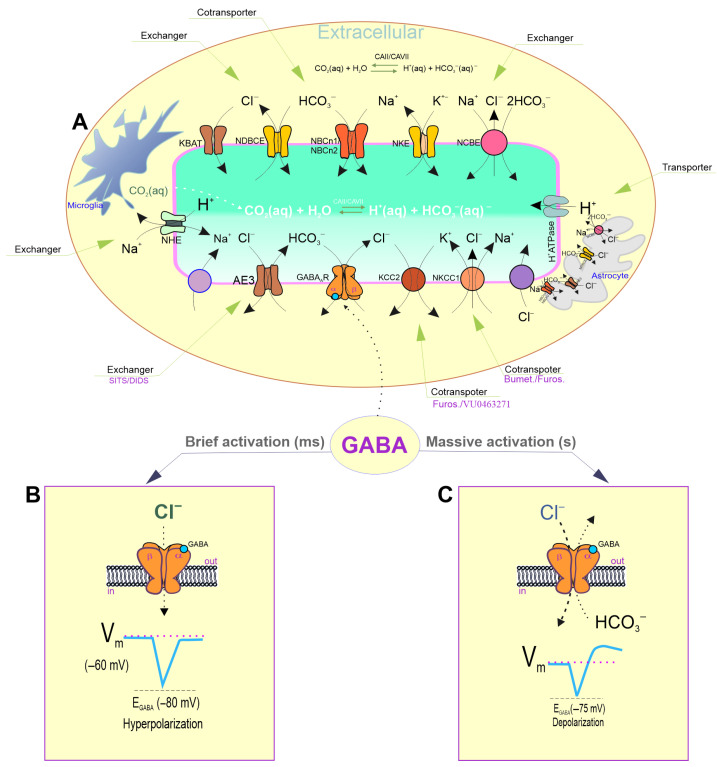
General diagram of the role of chloride and bicarbonate in GABAergic signaling. (**A**) Bicarbonate/pH and chloride homeostasis in neurons is maintained by membrane transporters and channels. Neuronal bicarbonate/pH levels are maintained by secondary active HCO_3_^−^ uptake via electro-neutral and electrogenic Na^+^/HCO_3_^−^ symporters (NBCN1/NBCN2), Na^+^/H^+^ exchangers (NHEs), and Na^+^-dependent/independent Cl^−^/HCO_3_^−^ exchanges (NCBE/NDCBE). The function of secondary active cation–chloride cotransporters (CCCs) and the activity of bicarbonate transporters are closely connected, and they are responsible for maintaining the neuronal Cl^−^ concentration ([Cl^−^]_i_) through K^+^ or Na^+^/K^+^-coupled cation transport. The correlation between the [HCO_3_^−^]_i_ and intracellular pH is directly linked to carbonic anhydrase activity, which quickly replenishes their intraneuronal level after changes. (**B**) After brief activation by agonist binding to the extracellular site in the structure, the receptors undergo conformational changes, resulting in pore opening and rapid Cl^−^ permeability, leading to hyperpolarization/inhibition of the *V_m_* in mature neurons. (**C**) During intense activation, GABAA receptors can be permeable to both Cl^−^ and bicarbonate ions. Bicarbonate ions, unlike Cl^−^, always flow out of neurons in the open state during such activation, resulting in depolarization/excitation of the *V_m_*.

**Figure 2:**
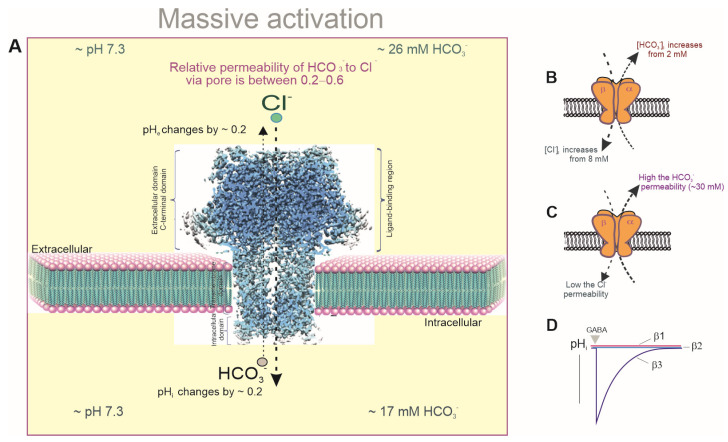
Neuronal bicarbonate/pH changes occur when GABA_A_ receptors are massively activated. (**A**) GABA_A_-mediated depolarization is connected to both a decrease in the intracellular bicarbonate concentration and a fundamental shift in the intra- and extracellular pH. (**B**,**C**) Models that hypothetically explain the ionic basis of GABA_A_R-mediated depolarization. (**D**) The homopentameric GABA_A_R β3 isoform, unlike the β1 and β2 isoforms, is capable of participating in GABA-mediated HCO_3_^−^ outflow from HEK 293 cells.

As members of the pLGIC family, γ-aminobutyric acid type A (GABA_A_R) receptors are the primary inhibitory receptors in the adult brain [7]. Nineteen different receptor subunits (α1–6, β1–3, γ1–3, δ, ρ1–3, ε, θ, and π) enable a variety of pentameric ensembles. The most prevalent receptor subtypes observed in the human brain have a 2α:2β:1γ composition [8,9,10]. After agonist binding with the ED of different GABA_A_R types [11], the receptors undergo conformational changes, causing pore opening followed by a rapid increase in the chloride ion (Cl^−^) permeability (Figure 1A). In mature neurons, this change leads to hyperpolarization/inhibition of the *V_m_* (Figure 1B) [12]. In addition to the hyperpolarization responses, GABA_A_Rs can induce the depolarization/excitation of the *V_m_*, not only during embryonic and early post-embryonic development [13,14] but also in adult brains (Figure 1C) [15,16,17]. In immature neurons, GABA-mediated depolarization is associated with a distinctive electrochemical gradient for Cl^−^, which is controlled by chloride transport systems (mainly secondary active cation–chloride cotransporters (CCCs)) [15,16]. This affects the direction and magnitude of the receptor’s effect on the neuronal excitability [17]. The depolarizing effects in mature neurons are associated with the formation of a high intracellular chloride concentration ([Cl^−^]_i_), which is observed during the massive (or intense) activation of the receptors by an agonist [18] or in neurodegenerative disorders, such as seizures [19,20]. During massive activation, the receptor pores become permeable not only to Cl^−^ but also to bicarbonate (HCO_3_^−^), which always flows out of neurons (Figure 1C) [21]. Such GABA_A_-mediated depolarization is associated with both a decrease in the intracellular HCO_3_^−^ concentrations ([HCO_3_^−^]_i_) and a fundamental shift in the intra- and extracellular pH, as shown in Figure 2A [22,23]. High HCO_3_^−^ (*E_HCO3−_*) equilibrium potential is a direct consequence of lower neuronal HCO_3_^−^ than extracellular HCO_3_^−^ levels [24] and is sustained through secondary active HCO_3_^−^ uptake via electroneutral and electrogenic Na^+^/HCO_3_^−^ symporters (NBCN1/NBCN2), Na^+^/H^+^ exchangers (NHEs), and Na^+^-dependent/independent Cl^−^/HCO_3_^−^ exchanges (NCBE/NDCBE) [25,26] (Figure 1A). However, the maintenance of the neuronal HCO_3_^−^ levels is directly correlated with the intracellular pH (pH_i_) through cytosolic carbonic anhydrase (CA) activity (Figure 2D), which rapidly converts CO_2_ into HCO_3_^−^ under a constant intracellular pH (Figure 1A) [27,28]. Thus, the opposing transport of Cl^−^ and HCO_3_^−^ through the receptor pores (Figure 2B,C) and the resulting decrease in the neuronal pH are essential factors that regulate GABAergic signaling during the massive activation of receptors in mature neurons (Figure 2A) [29]. In addition, the massive activation of GABA_A_Rs [30] can directly modulate the receptor states by altering desensitization and/or resensitization [31,32]. Indeed, over time, inhibitory receptors can become less responsive (desensitized) to GABA, but factors that prevent or delay this process can result in prolonged activation [31]. Studies conducted in recent decades have shown that prolonged receptor activation is coupled with phosphorylation mechanisms [33], the presence of Ca^2+^ and the state of receptor resensitization [34]. Specifically, the reduction in the neuronal ATP during massive GABA_A_R activation causes a decline in their functional activity, resulting in neuronal excitation. Different receptor subtypes have varying sensitivity to agonists (including GABA), and their subunit composition can influence their overall function [35]. Understanding these mechanisms is crucial for comprehending the role of various GABA_A_R subtypes in both normal brain function and neurodegenerative disorders.

The objective of this work is to analyze information on the relationship between the massive activation of inhibitory signaling and the ionic changes in neurons. The role of the massive activation of receptors in GABAergic depolarization and theoretical considerations regarding the mechanisms of chloride and bicarbonate ion permeability through ion channel pores are also discussed. This review summarizes the role of the transport systems involved in inhibitory synaptic plasticity and facilitating the restoration of the [Cl^−^]_i_ and [HCO_3_^−^]_i_/pH_i_ following GABA_A_R activity. Finally, the potential role of receptor dysfunction during massive activation and the resulting ionic and metabolic disruption in neurons during the manifestation of network/seizure activity are considered.

## 2. The “GABA Switch” from Inhibition to Excitation During Massive Activation

The GABA_A_-mediated single inhibitory postsynaptic potentials (IPSPs) can be hyperpolarizing, shunting or depolarizing under resting conditions [36,37]. Brief activation of dendritic GABA_A_R leads to hyperpolarization of the *V_m_* (Figure 1B), whereas the massive activation of dendritic GABA_A_Rs induces *V_m_* depolarization (Figure 1C) [38]. The massive activation of GABA_A_ receptors is a physiological event that initially leads to significant inhibition, followed by excitation of the nervous system. There are several factors that can cause this event. High concentrations of GABA in the extracellular space may be the main cause of massive activation. This may be due to increased synthesis, reduced reuptake, or disrupted degradation of GABA [35]. Furthermore, the use of GABAergic modulators such as barbiturates, benzodiazepines or neurosteroids can contribute to intensive receptor activation [31]. These drugs enhance the effects of low concentrations of GABA by binding to specific sites on the receptor structure and increasing the frequency with which the channel opens [12]. However, the effect of these ligands on depolarization depends on the concentration and the specific neural context: they can both induce and inhibit it. Specifically, benzodiazepines and pentobarbital can potentiate GABA_A_-mediated depolarization at low concentrations but mimic GABA’s depolarizing effect at higher concentrations [39]. Thus, GABAergic ligands have the capacity to modulate substantial receptor activation by GABA. Some studies have shown that the massive activation of dendritic GABA_A_Rs induces depolarization [15,40,41]. The dual nature of the GABA response involves a fast, initial hyperpolarizing component followed by a depolarizing phase that can last several seconds. This phase outlasts the action of any possible synaptically released mediator and is followed by prolonged epileptic afterdischarges that resemble seizures [35]. It is important to note that the depolarization is strong enough to trigger spike bursts in CA1 pyramids [42,43]. This demonstrates that the signaling role of GABA can change qualitatively from inhibitory to excitatory during intense interneuronal network activity [43]. For example, the dual GABA_A_R responses in hippocampal pyramidal neurons can be induced by the intensive activation of hippocampal interneurons [44]. Bonnet and Bingmann showed that the postsynaptic GABA_A_-mediated responses of cortical neurons consisted of early somatic hyperpolarization and dendritic depolarization [45]. The depolarizing responses can be seen in structures with low volume-to-receptor-density ratios and are easily elicited in dendrites but not in somas [46,47].

### 2.1. Changes in pH_i_ During Massive Activation

Early studies demonstrated that sustained GABA_A_R activation could lead to changes in the distribution of Cl^−^ and HCO_3_^−^ and a decrease in the pH_i_ from 7.3 to 7.1 (Figure 2A) [48,49,50,51,52]. In pyramidal neurons in adult neocortical slices with a more hyperpolarized resting membrane potential and low [Cl^−^]_i_, the HCO_3_^−^-mediated current component can exceed the GABA-mediated Cl^−^ current, resulting in HCO_3_^−^-dependent depolarizing IPSPs, as shown in some studies [41,53,54]. The authors showed that HCO_3_^−^ fluxes contributed to GABA-induced depolarization rather than to hyperpolarization, which was mainly driven by Cl^−^ flux. In a solution containing 30 mM HCO_3_^−^, the GABA-mediated current increase was associated with a depolarization of approximately 15 mV, an increase in the [Cl^−^]_i_, and a decrease in the [HCO_3_^−^]_i_. During the intensive activation of GABA_A_Rs, Cl^−^ inflow was shown to overwhelm KCC2-induced Cl^−^ extrusion, resulting in the collapse of homeostasis in neurons. In such situations, the HCO_3_^−^ current would predominate and drive the *V*_m_ toward the action potential threshold, which would result in GABAergic depolarization and excitation [16,24]. Further research confirmed that in pyramidal neurons in adult neocortical slices with a more hyperpolarized resting *V*_m_ and low [Cl^−^]_i_, the HCO_3_^−^-mediated current component could exceed the GABA-mediated Cl^−^ current and result in HCO_3_^−^-dependent depolarization [55,56]. GABA-evoked dendritic depolarization has a much slower rise time than either the hyperpolarizing response or the depolarizing response seen in neurons that maintain a depolarized resting Cl^−^ reversal potential (*E_Cl_^−^*) [21].

### 2.2. Maintaining pH_i_/Bicarbonate Homeostasis

The maintenance of the acid–base balance is ensured by homeostatic ionic mechanisms, which are of critical importance for all cells, including neurons and glia [57]. The typical resting or steady-state pH_i_ of a hippocampal neuron in CO_2_/HCO_3_-containing media is ~7.03–7.46, depending on the preparation, while the pH_e_ is ~7.35 (Figure 1A) [58,59]. The transmembrane transporters implicated in bicarbonate/pH regulation include Na^+^/K^+^ (NKE) and Na^+^/H^+^ exchangers (NHE), Cl^−^/HCO_3_^−^ exchangers, and Na^+^/HCO_3_^−^cotransporters (Figure 1A) [60,61]. The SLC4 family of Cl^−^/HCO_3_^−^ exchangers is one of the most well-known families of HCO_3_^−^ transporters and is widely expressed in cells [62,63]. The family comprises ten members (SLC4A1–5 and A7–11). Some of these members mediate Na^+^-independent Cl^−^/HCO_3_^−^ exchange (AE1-AE3), while others facilitate Na^+^-dependent Cl^−^/HCO_3_^−^ exchange (NCBE and NDCBE) [64]. AE transporters facilitate the extrusion of HCO_3_^−^ while simultaneously transporting Cl^−^ within the neuronal compartment. In contrast, NCBE and NDCBE facilitate the transport of HCO_3_^−^ into the cell and Cl^−^ out of the cell (Figure 1A). Cl^–^/HCO_3_^−^ exchangers have been demonstrated to play a pivotal role in maintaining the baseline pH_i_ levels and facilitating recovery following pH modifications [25]. Specifically, the process of acid extrusion, which typically increases the pH_i_, is achieved in neurons by the action of the SLC4 and SLC9 families of Na^+^-coupled transport proteins. The inwardly directed Na^+^ gradient established by Na^+^/K^+^-ATPase is utilized by both transporter families to either extrude H^+^ from the cell (in the case of Na^+^/H^+^ exchangers (NHEs)) or accumulate a weak base, such as HCO^3−^. [HCO_3_^−^]_i_ values of approximately 17 mM have been estimated [22]. Excessive alkalosis, which is probably a rather exceptional event under physiological conditions, leads to the activation of the Na^+^-independent anion exchanger (AE3), which extrudes base equivalents in the form of HCO_3_^−^ in exchange for Cl^−^ [65,66]. AE3 mediates the countertransport of one Cl^−^ with one HCO_3_^−^, leading to the depletion of [Cl^−^]_i_ and Cl^−^ accumulation in the extracellular medium [25]. The role of Na^+^-dependent Cl^−^/HCO_3_^−^ antiporters in regulating Cl^−^ extrusion to maintain neuronal [Cl^−^]_i_ homeostasis is not fully understood [67]. Neuronal acid extrusion is mainly achieved by Na^+^-driven Cl^−^/HCO_3_^−^ exchange (NDCBE) and Na^+^/H^+^ exchange [64] (Figure 1A). Both types of transporters are activated by a decrease in the pH_i_, which can be caused by GABAergic transmission; for example, leading to a channel-mediated net efflux of HCO_3_^−^.

Buffering by CO_2_/HCO_3_^−^ is one of the most important mechanisms of tissue pH control. The presence of a dynamic equilibrium between CO_2_ and H_2_O and the presence of H^+^ and HCO_3_^−^ is a key feature of this system [68]. This equilibrium is quickly reached through the action of enzymes from the CAs family (see Figure 1A). Carbonic anhydrases catalyze the reversible hydration of CO_2_ to H_2_O and HCO_3_^−^. Twelve catalytically active CA isoenzymes are known, five of which show cytosolic localization [69]. Bicarbonate buffering and carbonic anhydrase activity protect brain tissue from acidification by converting H^+^ and HCO_3_^−^ into H_2_O and CO_2_. The resulting CO_2_ is then removed by the cerebral circulation and transported to the lungs for exhalation. Therefore, to maintain the stability of the brain tissue pH, there is a need for an effective mechanism that can supply HCO_3_^−^ to the extracellular space in a way that is responsive to neuronal activity. The rapid replenishment of [HCO_3_^−^]_i_ by the activity of cytosolic CA isoforms occurs during a large GABA_A_-mediated net efflux of HCO_3_^−^ [70]. Thus, the net extrusion of acid from neurons and glial cells is achieved by secondary active transport in which the efflux of H^+^ or influx of HCO_3_^−^ is coupled with Na^+^ influx using energy stored in the transmembrane Na^+^ gradient. Several acid–base-coupled carriers are also involved in the transport of metabolites, including lactate and amino acids. Lactate transport via monocarboxylate transporters has been suggested to play an important role in the energy supply of neurons, leading to the “astrocyte-to-neuron lactate shuttle hypothesis” [71]. The major acid–base transporters identified in astrocytes include the electrogenic sodium–bicarbonate cotransporter 1 (NBCe1) encoded by the SLC4A4 gene, the sodium–hydrogen exchanger (NHE1) encoded by the SLC9A1 gene, the sodium-dependent chloride-bicarbonate exchanger (NCBE) encoded by the SLC4A10 gene, the chloride–bicarbonate exchanger (AE) encoded by the SLC4A1 gene, a proton pump, and vacuolar V-ATPase (Figure 1) [72]. Moreover, astrocytes express both intra- and extracellular CA isoforms, with carbonic anhydrase II (CAII) being particularly abundant. Of the various cells involved in the synaptic neuropil, astrocytes appear to be well suited to actively controlling the local brain pH_e_ microenvironment.

### 2.3. Changes in [Cl^−^]_i_ During Massive Activation

A detailed analysis of the ionic basis of the GABA_A_R-mediated biphasic responses indicated that the initial part of the depolarization was caused by anionic redistribution in which the inward HCO_3_^−^ current drove a depolarization that promoted Cl^−^ uptake and hence caused a positive shift in the *E_GABA_* [21,23,43]. Such studies demonstrated that neuronal transmembrane Cl^−^ transport is rate-limited. Significant changes in the dendritic [Cl^−^]_i_ and *E_Cl_^−^* occur because of intense GABA_A_R activation, and the transmembrane gradients for Cl^−^ in somas and HCO_3_^−^ in dendrites are significantly less affected by large GABA_A_R-mediated Cl^−^ currents. Several theoretical models have been proposed to explain the ionic basis of slow post-tetanic GABA_A_R depolarization. Specifically, the GABA_A_R-induced depolarization potential has been shown to be dependent on the preservation of HCO_3_^−^ and the collapse of the Cl^−^ gradients (Figure 2B) [21]. The key argument of the gradient collapse hypothesis is that influx of chloride ions through the receptor pore would lead to an increase in the [Cl^−^]_i_, thereby shifting the *E_GABA_* in a positive direction. Experiments using the massive activation of dendritic GABA_A_Rs demonstrated that synaptic Cl^−^ influx through the ion channel pore could exceed the maximum transport rate. This hypothesis is reasonable, given that pH alterations would accompany dissipation of the HCO_3_^−^ gradient at a constant pCO_2_, while the intracellular pH would be 6.3 if the *E_HCO3_^−^* was driven to the *V_m_*, a process that would cause protein denaturation and neuronal damage. To avoid this type of damage, the neuronal HCO3- concentration is stabilized via the diffusion and hydration of CO_2_, while the pH_i_ is supported by intracellular buffers and H^+^ efflux. Opposing HCO_3_^−^ and Cl^−^ flux through ionic channel pores resulted in a reduction in the electrochemical gradient that drove the hyperpolarizing Cl^−^ inflow, thereby allowing the depolarizing HCO_3_^−^ outflow to prevail. These studies provided estimates of the maximum transport rates for Cl^−^ of 5 to 7 mM/s and indicated that the *V_max_* could be exceeded by GABA_A_R-evoked Cl^−^ inflow into neurons. The authors reported that during depolarization, the [Cl^−^]_i_ increased from 8 mM, whereas the [HCO_3_^−^]_e_ increased from 2 mM (Figure 2B) [15]. The Cl^−^ accumulation model is attractive because it could explain why the enhanced synaptic release of GABA is required to elicit depolarizing responses. However, an argument against the Cl^−^ gradient collapse hypothesis is that the stability of the HCO_3_^−^ gradient appears to require both extra- and intracellular CA function. However, benzolamide, an enzyme inhibitor that is confined to the extracellular space, does not block the depolarizing GABA responses [21].

An alternative model contradicts the Cl^−^ accumulation hypothesis and suggests that distinct GABA_A_R subtypes with special permeability for anions are present, namely lower Cl^−^ and higher HCO_3_^−^ permeability through ion channel pores and slower kinetics. However, the authors were unable to identify these receptor subtypes [40,41]. In addition, when the intracellular anion concentrations were manipulated and the polarity of the giant biphasic GABA-mediated postsynaptic current was reversed, neuronal Cl^−^ accumulation did not occur during the early inward current. Thus, this result does not explain why an inward Cl^−^/HCO_3_^−^ current would switch to an outward one. The authors suggested that for neurons with a high [Cl^−^]_i_, the Cl^−^ in the cell would be depleted during the early (inward current) part of the response, which would have to occur at a rate comparable to that of the Cl^−^ accumulation, and the polarity of the late response would be determined by the HCO_3_^−^ gradient. Recent molecular studies support the existence of GABA_A_R subtypes with high HCO_3_^−^ permeability, which could be responsible for the depolarizing GABA_A_ responses. Only the homopentameric GABA_A_R β3 isoform, in contrast to the β1 and β2 isoforms, was shown to participate in GABA-mediated HCO_3_^−^ outflow from HEK 293FT cells (Figure 2D) [73].

### 2.4. Maintaining [Cl^−^]_i_ Homeostasis

**Figure 3:**
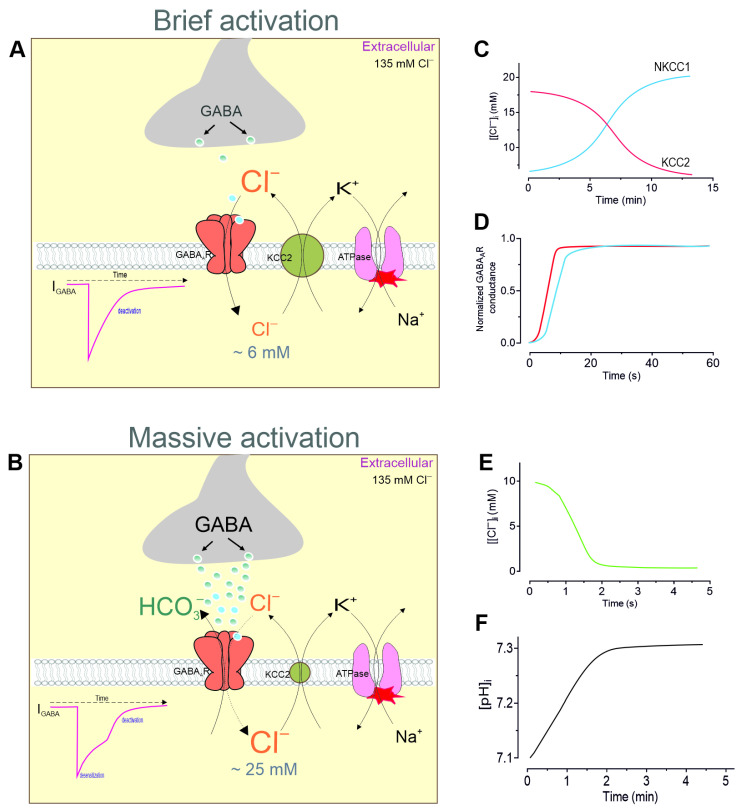
Inhibitory synaptic plasticity during brain development. (**A**) A diagram showing the Cl^−^ influx through the ion channel pore during the brief activation of GABA_A_Rs and the main structures that hyperpolarize the V_m_ in mature neurons. (**B**) A diagram illustrating the Cl^−^ influx and HCO_3_^−^ outflow through the channel pore during GABA_A_Rs’ massive activation, and the primary structures responsible for the V_m_ depolarization in mature neurons. (**C**) Time course of the [Cl^−^]_i_ recovery through NKCC1 and KCC2 activity. (**D**) The recovery of GABA_A_R conductance following massive activation. (**E**) The time required for the recovery of the [Cl^−^]_i_ after receptor activity. (**F**) The time required for pH recovery following receptor activity.

The neuronal anion concentrations are key parameters controlling the E_GABA_; their effect depends on the activity of the neuronal network and the stage of neuronal development [73,74]. Although neuronal chloride homeostasis is determined by several Cl^−^-conducting systems [75], secondary active cation–chloride cotransporters (CCCs) play a key role in maintaining the [Cl^−^]_i_ (Figure 3A) [76]. CCCs are members of the *SLC12A* nine-member gene family, which have a common evolutionary origin and include four K^+^/Cl^−^ cotransporters (KCCs), two Na^+^/K^+^/2Cl^−^ cotransporters (NKCCs), an Na^+^/Cl^−^ cotransporter (NCC), and two cotransporters, CCC9 and CIP [77]. CCCs are large glycoproteins (∼110–130 kDa) with 12 transmembrane segments and intracellular amino- and carboxy-terminal domains [78]. Two members of the SLC12A family, NKCC1 and KCC2, which are expressed in the vertebrate brain and whose activity determines the switching of GABAergic signaling from excitation to inhibition, are of particular interest [17]. CCCs do not consume ATP directly but use the energy stored in the ionic gradients established by Na^+^/K^+^-ATPase to transport other ions, including Cl^−^, against their electrochemical gradients. NKCC1 mediates Cl^−^ uptake driven by the intracellular Na^+^/K^+^ gradients (Figure 3A), whereas KCC2 extrudes Cl^−^ under physiological conditions using the K^+^ gradient (Figure 3B) [79,80]. NKCCs are activated by phosphorylation processes and KCCs are activated by dephosphorylation [81]. The phosphorylation pathway that targets NKCC is repressed by intracellular [Cl^−^], leading to an inhibitory negative feedback loop when the intracellular [Cl^−^]_i_ reaches a sufficiently high level.

Progress in understanding the contribution of specific CCCs to GABAergic signaling has been made possible using blockers of these chloride transport systems. Specifically, the Cl^−^ gradient was shown to be reduced by the loop diuretic furosemide and to depend on the extracellular K^+^ [82], which together revealed a distinct neuronal K^+^/Cl^−^ transport process. The net transport activity of KCC2 depends on the chemical gradients of K^+^ and Cl^−^ and is inhibited by the diuretic furosemide [68]. When adults are treated with furosemide, there is a greater increase in the [Cl^−^]_i_ than when treated with P10 cells. This indicates that KCC2 activity rises with age (see Figure 1A) [83]. Although furosemide is often used to block KCC2, this drug also effectively blocks NKCC1 [84,85]. Selective KCC2 blockers (e.g., VU0463271) currently exist, while another diuretic, bumetanide, selectively blocks depolarizing GABAergic responses in immature pyramidal neurons and is considered to be a specific NKCC1 blocker [86,87]. However, despite many molecular biological studies demonstrating the involvement of CCCs [88,89,90], some studies have questioned their role in the rapid recovery of chloride homeostasis after receptor activity [22,91]. The time constant for neuronal chloride homeostasis recovery after a [Cl^−^]_i_ increase of about 10 mM was shown to be more than 5 min (Figure 3C) [92]. Another study showed that the KCC2-mediated extrusion of ~5 mM Cl^−^ required more than 5 min after furosemide-induced [Cl^−^]_i_ changes [83]. Bumetanide-sensitive NKCC1-mediated Cl^−^ uptake in immature rat neocortical neurons and human neuroblastoma cells was observed over a time interval of 5–15 min (Figure 3D) [93,94]. Gonzalez-Islas et al. demonstrated that Cl^−^ recovery following depletion in chick embryonic motoneurons exhibited both NKCC1-independent (1–10 min) and NKCC1-dependent (10–50 min) properties [95]. In contrast, the recovery of GABA_A_R conductance following desensitization exhibited a time constant of a few seconds (Figure 3D). Specifically, the recovery of GABA-evoked conductance after desensitization in different neurons had a time constant of approximately 13 s [96,97], while the time course of [Cl^−^]_i_ recovery (Figure 3E) inferred from the I_GABA_ series was several seconds and the restoration of the pH_i_ took about 1 min (Figure 3F). In addition, in vivo studies of mouse pyramidal neurons revealed that an increase in the [Cl^−^]_i_ of 12 mM, which occurred following epileptic activity, was recovered in under 30 s [98].

## 3. Conformational Changes During Massive Activation

**Figure 4:**
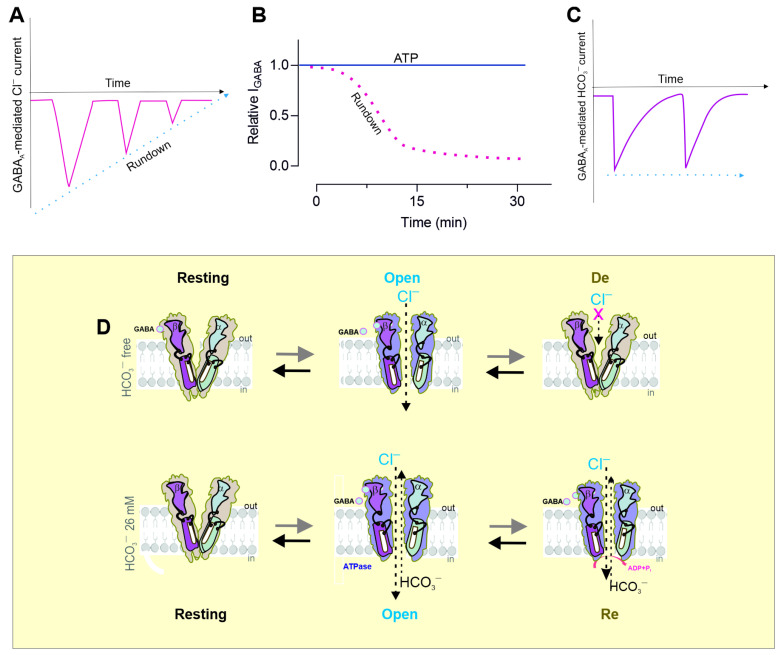
Bicarbonate and ATP/ATPase in GABA_A_R function. (**A**,**B**) The rundown effect of the receptor function during massive activation or long-term potentiation, respectively. The rundown effect of GABA_A_R activity is not observed when Mg^2+^ATP (2 mM) is present. (**C**) The rundown effect of the receptor function is not significantly manifested in the presence of HCO_3_^−^ (~26 mM). (**D**) A model illustrating the four basic conformational states that represent GABA_A_R function, which include a resting state, an open state, a desensitization state, and a resensitization state. In an HCO_3_^−^-free experimental medium, the first application of GABA shifted the equilibrium from the resting state to the high-affinity open state, which then entered a state of desensitization. In contrast, in the presence of HCO_3_^−^ (~26 mM) in an experimental medium, GABA application induced changes in receptor conformation and shifted the equilibrium from the resting state to the high-affinity open state; here, agonist application resulted in receptor resensitization. Bicarbonate preferentially stabilized the receptor structure.

After GABA is released from vesicles, synapse receptors are activated by the brief or intensive action of an agonist. As a result, GABA_A_Rs undergo conformational changes that result in channel opening and GABAergic mIPSC induction [99]. As the GABA concentration changes are transient and short, the IPSC decay reflects the closure of channels following ligand removal, namely deactivation [34,100]. Some studies demonstrated that exposure to micromolar GABA concentrations could lead to receptor activation, the generation of sustained low-amplitude current, and the conversion of the receptor into a desensitized state [31]. Currently, four basic types of receptor function states during sustained or repeated agonist binding have been demonstrated: resting, open, desensitized, and resensitized (Figure 4D) [101,102,103,104]. GABA_A_Rs’ function is to pass through the series of states described above. The time spent in each state and, thus, the time course of the postsynaptic current are determined by the receptor’s properties and the agonist exposure profile [105,106,107]. Specifically, the expression of different receptor subtypes contributes to differences in the IPSC decay observed at various stages of development [108] and in different cell types [109]. Typically, IPSCs last considerably longer in immature neurons than in mature neurons. For example, currents activated by brief pulses of (>1 µM) GABA to outside-out patches from cultured hippocampal neurons were found to mimic GABA-mediated IPSCs, during which channels enter lengthy closed states and then subsequently reopen. Movement through these states was shown to result in paired-pulse desensitization [110]. This high-affinity state was found to disappear upon agonist removal [106]. However, [^3^H]GABA dissociates four-fold faster than functional recovery, indicating that desensitized receptors need not be bound by an agonist. Following the application of 1 µM GABA, approximately 5% of the α1β3γ2L subtype was found to have been functionally eliminated due to desensitization [111].

Many receptor desensitization studies have focused on the changes to receptors following massive activation with a neuromediator or allosteric drug that affects the occupancy of this state during synaptic inhibition [112,113]. Such changes may include the phosphorylation of receptor subunits by kinase activity [31] and changes in expression, clustering [114], and pharmacological selectivity. Some authors have shown that the cross-desensitization of GABA_A_R-mediated Cl^−^ currents is evoked by changes in the [Cl^−^]i [96]. In contrast, other studies demonstrated that the extent of the desensitization, as assessed by the steady-state-to-peak ratio, was significantly greater at a high intracellular ([Ca^2+^]_i_) [115]. Recovery from desensitization was measured at 30 s intervals using a double-pulse protocol and found to be significantly slower at a high [Ca^2+^]_i_ than at a low [Ca^2+^]_i_ [116]. Receptor mutants and allosteric modulators were used to demonstrate that the desensitization of GABA_A_Rs facilitated their phosphorylation by protein kinase C (PKC), as well as increases in the number of receptors at inhibitory synapses. These observations provide physiological relevance for the desensitized state, which may act as a signal regulating the efficacy of inhibitory synapses during prolonged periods [31].

Structural studies have demonstrated that differences between the agonist-bound open state and the desensitized state are conformational changes that occur at the inner face of the receptor. However, recent functional and structural studies provide compelling evidence for a dual-gate model in which the TMD of pLGICs contains both an activation gate, which is in the upper half of the channel, and a de-gate, located on the intracellular side. Some authors have reported a very diverse and labile conformational landscape during desensitization after introducing mutations in different subunits [3]. Kinetic modeling indicated that the subunits could move independently, with the desensitization of two subunits being necessary to occlude the pores. These findings are consistent with the results of other studies. For example, differences in the conformational properties of the activation and desensitization states of GABA_A_Rs were identified using the sulfhydryl alkylating reagent n-ethylmaleimide (NEM) [104]. In particular, the important role of the conformational rearrangements and the resulting changes in the phosphorylation and dephosphorylation processes were confirmed using a mutant receptor that produced a significant alteration in the receptor’s capability to access the transition from a desensitized to a resensitized state. Receptor desensitization strongly correlates with the slowing of its deactivation, namely, resensitization [115]. The correlation between the desensitization and resensitization of GABA_A_R-mediated currents can be seen more clearly in response to prolonged or repeated mediator application, which allows more desensitization rather than a short pulse (Figure 4A) [113]. In particular, the reapplication of 100 μM GABA did not result in the desensitization of GABA_A_R-mediated HCO_3_^−^ (Figure 4C). Recently, fluorescence studies also demonstrated that GABA_A_R-mediated HCO_3_^−^ outflow in the presence of HCO_3_^−^ did not occur after a second application of the agonist; instead, only resensitization occurred [103]. Furthermore, the recovery of the [HCO_3_^−^]_i_ after receptor activity, which appears to occur at ~30–40 s, was consistent with data on the recovery of GABA_A_R-mediated conductance after desensitization at ~40 s [22,96,104]. Thus, although the regulation of GABA_A_R desensitization and resensitization is an important mechanism controlled at the receptor level and by its signaling pathway [31], such regulation may be important in understanding the role of HCO_3_ in receptor activity. It is more likely that the change in the [HCO_3_^−^]_i_ is likely the major determinant of the onset of receptor resensitization, whereas the change in the [Cl^−^]_i_ in bicarbonate-free medium plays a critical role in desensitization.

## 4. Modulation of Desensitization by pH

Recent works have shown that pH alterations and the membrane potential affect GABA_A_R gating, mainly via modulation in terms of agonist binding and receptor desensitization. Their combined actions were investigated to test whether the effects of the membrane potential and pH are additive [117]. An examination of the existing responses to rapid GABA applications revealed that the current–voltage relationship was almost straight at an acidic pH. Nevertheless, an increase in the pH induced inward rectification. The process of desensitization was found to be enhanced at depolarizing potentials, although this depolarization was observed to be pH-dependent, with a weaker effect observed at an acidic pH and a stronger effect at a basic pH. A comparable pattern was identified with regard to the onset of responses to saturating GABA concentrations. Additionally, extracellular H^+^ was demonstrated to allosterically modulate GABA_A_Rs. Specifically, the modulation of GABA_A_Rs by H^+^ was shown to generate a synaptic GABA transient, highlighting the importance of the desensitization process [118]. In the case of the α1β2γ2 and α1β2 receptor subtypes, the application of H^+^ notably affected the current time course characterized by a reduction in the binding and desensitization rates [119]. The amplitude of the α1β2 receptor-mediated currents was found to be significantly affected by protons, while the effect on their kinetics was observed to be relatively limited. This finding indicates that the primary mechanism is likely to be direct, noncompetitive inhibition, with a lesser contribution from allosteric regulation. The evidence demonstrates that extracellular H^+^ has a significant impact on GABA_A_Rs.

## 5. Metabolic Changes During Massive Activation

Early studies of the GABA_A_R properties in hippocampal neurons [33], cultured chick spinal cord neurons [120], dissociated nucleus tractus solitarii [121], isolated bullfrog dorsal root ganglion neurons [122], cerebellar granule cells [123], rat pyramidal neurons [124], and hypothalamic hamartomas [125] demonstrated that the GABA-mediated Cl^−^ conductance decreased progressively with the time (maximum effect at 12 min) (Figure 4B). The inactivation of GABA_A_Rs could be prevented by the concomitant inclusion of ATP and Mg^2+^ at concentrations of ≥2 mM, indicating that these chemicals are necessary to prevent the inactivation of the GABA response [33]. The GABA_A_ Cl^−^ current was also maintained by the addition of 2 mM ADP or ATPγS (adenosine 5′-O-3-thiotriphosphate) to an internal medium containing Mg^2+^ [124]. The nonhydrolyzable ATP analog β,γ-imidoadenosine-5′-triphosphate did not produce any effects [125]. It appears that the maintenance of GABAR function is linked to an ATP-dependent process, which is likely to be a phosphorylation event. The magnitude of the rundown was comparable in both recombinant α1β2γ2 and β2γ2 receptors [126]. Neither the stimulation nor the inhibition of protein kinase A or C significantly affected the rundown. However, the addition of sodium vanadate, an inhibitor of protein tyrosine phosphatase, led to a significant reduction in the rundown. In addition, inhibiting protein tyrosine kinase activity by either genistein or lavendustin A induced the rundown of the GABA response. The authors presented the hypothesis that an ATP-sensitive binding site exists on the cytoplasmic side of the cell membrane and that receptor activity may entail not only ATP binding but also ATP hydrolysis mechanisms [33,121,127]. Additionally, hypoxia was shown to cause the depression of GABA-mediated IPSCs in the CA1 neurons of hippocampal slices [124,128].

## 6. Ionic Changes During Network/Seizure Activity

Dynamic functional brain connectivity plays a pivotal role in enabling adaptive cognition and behavior. Abnormal changes in this connectivity can lead to dysfunction, which is common in a range of neurological conditions. Epilepsy is one of the most common neurological disorders, defined by the seemingly random occurrence of spontaneous seizures [129,130,131]. The epileptic brain network is made up of a set of cortical and subcortical brain regions that are structurally and functionally connected. These regions are distributed across lobes and hemispheres, and their connections and dynamics evolve over time [132]. A range of epileptic manifestations, including focal and generalized seizures, were proposed to originate from, propagate through and terminate at network nodes and edges, which also generate and maintain normal physiological brain dynamics. Network-based approaches can help us to gain a deeper understanding of the mechanisms that underlie the emergence of seizures in evolving epileptic brain networks [133]. Considering the above, concerted experimental and theoretical efforts have been made to elucidate the underlying mechanisms of seizure onset and propagation [134,135,136]. Understanding more about these mechanisms may help in developing more effective therapeutic interventions [137]. However, the current technical problems make it challenging to study their specific contribution in an experimental setting [22].

**Figure 5:**
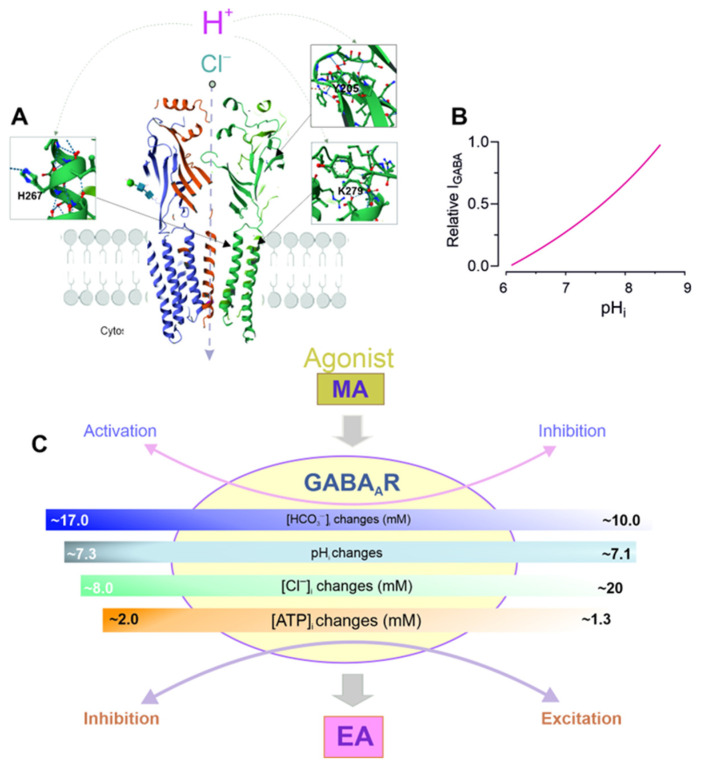
Massive GABA_A_R activation and network/seizure activity. (**A**) Sites of H^+^ binding with the GABA_A_R β subunit (Protein Data Bank, 4COF). (**B**) Relative I_GABA_ as a function of the pH_i_. (**C**) The diagram illustrates how receptor massive activation (MA) affects the [HCO_3_^−^]_i_, [Cl^−^]_i_, pH_i_, and [ATP]_i_, leading to the appearance of epileptiform activity (EA).

The synchronization of neuronal activity is said to be fundamentally dependent on GABAergic neurotransmission, according to experimental and theoretical evidence [138]. The role of GABA_A_Rs in epileptiform activity (EA) has received increasing attention, in part because GABA-induced Cl^−^ currents can be depolarizing and, thus, excitatory under certain circumstances [139]. Furthermore, as described in previous sections, during prolonged GABAergic stimulation (e.g., epileptic seizures), the stable depolarizing drive of GABA_A_R-mediated HCO_3_^−^ currents will enhance the activity-dependent Cl^−^ inflow into neurons and, thus, directly contribute to the generation of excitation under these conditions [18,140,141]. For example, depolarizing shifts in the *E*_*GABA*_ of the pyramidal neurons in the hippocampus are caused by intense receptor activation during an epileptiform discharge [142,143,144], which can switch their action such that they begin to promote rather than oppose EA [145]. However, such [Cl^−^]_i_ dynamics coupled with EA coincide with other changes, including increases in intracellular H^+^ (i.e., acidification) and reductions in extracellular H^+^ (Figure 5A) [146,147].

Specifically, whole-cell recordings from stratum interneurons and CA1 pyramidal cells demonstrated that some GABA-mediated currents were depolarizing during EA and were suppressed by CA blockers or activators [148,149,150]. Fluorescence and optical genetic methods have demonstrated a clear increase in the dynamic [Cl^−^]_i_ and pH_i_ changes during neurological disorders [151,152,153] and increased network activity (Figure 5B). For example, during epileptiform discharge, all the GABAergic interneuron subtypes converge on elevated [Cl^−^]_i_ [154,155,156]. In parallel with these dynamics, EA leads to varying degrees of intracellular acidification, reflecting the baseline pH [146,157]. Călin and coauthors demonstrated that similar to pyramidal neurons, SST and VIP interneurons tended to accumulate [Cl^−^]_i_ during EA, whereas the neuronal chloride levels remained unchanged in the PV interneuron populations during EA [158]. Chloride imaging data allowed the authors to suggest that the GABAergic synaptic input to the different neuronal subtypes exhibits different chloride-dependent changes during EA [159]. These differences are unlikely to reflect EA-associated damage, as the ion concentrations recovered in the neuronal populations following each EA. Rather, the degree of loading across the subtypes appeared to be inversely related to the baseline chloride levels, suggesting that the initial driving force of GABA_A_Rs affects the degree of the EA-associated chloride shift. This is consistent with the observation that all the neuronal subtypes converged toward a common, high [Cl^−^]_i_ of approximately 20 mM during intense periods of EA [158]. Moreover, intracellular acidification was observed during EA, whereas the intracellular pH recovered between seizures.

## 7. Role of pH in GABA_A_R Modulation

The neuronal pH is determined by the equilibrium between the rate of acid influx/generation or alkali outflow/consumption and the rate of acid influx/consumption or alkali influx/generation [160]. The maintenance of a steady-state pH is the result of a balance between these processes [25,61]. The concentration of free H^+^ in neurons is found to be in the nanomolar range. The high buffering capacity of the cells provides a reservoir of acid equivalents in the millimolar range. The changes in the extracellular pH (pH_e_) accompanying physiological and pathological processes are sufficient to affect a number of important structures, such as ion channels, transporters and receptors. However, although several reports indicate that inhibitory signaling is strongly modulated by increasing or decreasing the pH [161,162,163] (Figure 5B), the physiological significance of the pH in the modulation of GABA_A_R activity is not fully understood. The authors hypothesized that the presence of H+ would affect the apparent binding affinity for receptors. Indeed, a significant shift in the EC50 for GABA was observed when the pH was adjusted to an acidic state, without any substantial impact on the maximal currents or the Hill coefficient [164]. Furthermore, an increase in the pH to an alkaline level was found to increase the frequency of channel opening and reduce the duration of the long-closed state, thereby raising the open probability [161]. Conversely, when the channels were exposed to an acidic pH, the outcome was the opposite: a decrease in the open probability. Other electrophysiological studies have shown that a pH (6.4) decreases and a pH (8.4) increases GABA-mediated chloride current in rat hypothalamic neurons and in α3β2γ2 GABA_A_R isoform stably expressed in HEK 293 cells [161,165,166]. The inhibitory effect of an acidic pH on the agonist response is reversible and concentration-dependent, resulting in a notable increase in the EC_50_ without a substantial change in the slope or maximal currents induced by GABA in the α1β2γL and α1β2 receptors [166]. In addition, H^+^ competitively inhibits the bicuculline-induced antagonism on mediator effects in the α1β2**γ**L receptors. Other authors have also demonstrated that, within the pH range of 5.0–9.0, an increase in the extracellular pH enhances, while a decrease suppresses, the GABA-mediated Cl**^−^** current in rat primary sensory neurons [167], as well as in freshly isolated rat dorsal root ganglia neurons, in a reversible and concentration-dependent manner (Figure 5B) [168]. The IC_50_ for these neurons was 7.1 pH. By contrast, several studies have demonstrated that the GABA-mediated chloride currents in rat cerebellar granule cells (CGCs) increase at an acidic pH of 6.4 and decrease at a basic pH of 8.4 [169,170]. The rectification of chloride channel conductance is associated with the protonation of base amino acids at an acidic pH. For example, in 6–12-day-old rat hippocampal CA3 neurons, it was demonstrated that increasing the extracellular pH to 8.5 or reducing it to 6.0 markedly enhanced or diminished the frequency and amplitude of spontaneous GABA_A_R-mediated postsynaptic currents [171].

## 8. Role of β3 Subunit in pH_i_ Changes and Seizure Activity

Several studies have shown that the impact of H^+^ on the functional activity of the GABA_A_Rs depends on the subunit composition. Although it is accepted that protons can control the probability of ion channels opening, the specific amino acid residues and receptor subunits that are responsible for H**^+^**-mediated modification are not well understood (see Figure 5A). To illustrate this, an elevation in the pH resulted in a reduction in the amplitudes of the currents evoked by saturating [GABA] for the α1β2γ2 and α1β2 subtypes. However, this effect was more pronounced for the α1β2 isoforms [119]. At pH 6.4, GABA activation of the αβγ receptors was enhanced at low GABA concentrations. A further decrease in the pH to 5.4 inhibited the GABA_A_R-mediated responses via αβγ receptors, while the mediator responses via αβ isoforms were enhanced. Inserting homologous β-subunit amino acid residues into the γ2 subunit in order to recreate the H^+^ modulatory profile of the αβ and αβγ receptor isoforms revealed that, in the presence of β2H267, the γ2T294K mutation is necessary in order to enhance the mediator response at pH 5.4 (Figure 5A). This residue, T294, is structurally similar to K279 in the β subunit. The authors suggested that the presence of a lysine at this position plays a key role in mediating the allosteric effects of changes in the pH, rather than forming a direct site for protonation within the GABA_A_R [29]. Replacing all the conserved external histidine residues with alanine on the β subunits demonstrated that H267, which is located in the TM2 domain, plays a key role in regulating H^+^ passage activity. The contrasting functional impacts of Zn**^2+^** and H**^+^** on this single histidine residue are probably due to differences in the charge delocalization on the imidazole rings within the receptor structure. Additional replacement of H267 in the β subunit with charged side-chain residues (such as glutamate and lysine) shows that this region of the ion channel significantly affects the functional properties of receptors [172]. A comparable report found that site-specific mutations of βY205 to F or α1F64 to A—both of which affect agonist binding—resulted in a notable decline in the pH sensitivity of the GABA response [164]. Other research found that the mediator response was reversibly and concentration-dependently inhibited by an acidic pH. A significant increase in the EC50 was observed without any notable change in the slope or maximal currents induced by GABA at the α1β2γ2L and α1β2 isoforms [166]. It is worth noting that H^+^ was effective in counteracting the bicuculline-induced antagonism of the agonist in the α1β2γ2L isoforms. The homomeric β3 subunit has recently been identified as an H^+^-gated anion channel [173]. Specifically, it has been demonstrated that a single H267A mutation in the β3 subunit completely eliminates channel activation by protons. Molecular dynamics simulations of the β3 crystal structure revealed that protonation of H267 enhances the number of hydrogen bonds formed between the H267 and E270 of the neighboring subunit. This resulted in the formation of a pore-stabilizing ring and Cl^−^ accumulation within the transmembrane pore. The activation of GABA-mediated Cl^−^/HCO**_3_**^−^ currents at physiological pH changes (pH_50_ is in the range 6.0–6.3) and kinetic studies suggest a physiological role in neuronal and non-neuronal tissues that express the β3 subunit [173].

Numerous works have emphasized the pivotal role of the β3 subunit in shaping the functional characteristics of GABA_A_Rs [174]. Specifically, β3 is the only subunit that determines the ionic selectivity of the recombinant receptor and can form a functional homomeric ion channel pore [175]. Furthermore, only the β3 subunit can fully maintain or restore receptor activity to control levels in the hippocampus when the β1–β2 subunit is knocked out [176]. The β3 subunit is also widely expressed in the immature and adult brain in circuits involved in seizure generation, such as the cortex, hippocampus, and thalamic reticular nucleus [177,178]. Genetic variants associated with developmental and epileptic encephalopathies have been identified in the GABRB3 gene, which is responsible for encoding the β3 subunit. The absence of the β3 subunit results in a lower production of functional receptors in null-β3 mice. Pharmacological evidence indicates that this reduction is not compensated for by other β subunits. Recent research has revealed that gain-of-function GABRB3 variants can increase or decrease the properties of receptor desensitization and that there is a correlation with the degree of disease severity [179]. Variants that reduced desensitization at equilibrium were found to be clustered within the transmembrane regions that constitute the channel pore, and they were found to be associated with greater disease severity. Conversely, variants that accelerated current decay were found to be clustered within the coupling loops responsible for receptor activation, and they were also found to be associated with lower severity.

## 9. Conclusions and Future Directions

Although receptor pores were shown to be permeable not only to chloride but also to bicarbonate ions over 30 years ago, the role and mechanisms of ionic changes during GABAergic depolarization remain somewhat mysterious. Bicarbonate is involved in the GABA_A_-mediated current only in the adult brain and under certain conditions. Here, we showed that the massive activation of GABA_A_Rs and the resulting ionic and metabolic changes in neurons can be considered to be a single pool of interrelated events (Figure 5C). Furthermore, the analysis of the data presented in this review suggests that if bicarbonate is involved in GABAergic depolarization/excitation, it may be the primary cause of these changes in neuronal metabolism. These changes are likely to occur because of the dysfunction of the β3-containing receptor subtypes. Furthermore, protons can directly regulate receptor function by modulating desensitization or resensitization. These results are consistent with other studies. For example, HCO_3_^−^ may modulate neuronal activity in a potentially pH-independent manner through the allosteric modulation of intracellular messengers. Nevertheless, it is crucial to recognize that no current, objective analytical compartment can be used to study and anticipate the heterogeneity associated with the propagation dynamics of seizure activity. Here, we demonstrate the β subunits to be important targets for the proton modulation of receptor function and drivers of a cascade of molecular events leading to the manifestation of neurodegenerative disorders. The analysis of the relationship between these processes could be useful in understanding the physiological and pathological processes in the brain. However, clear insight into the mechanisms of network or seizure activity requires the detailed structural analysis of the conformational rearrangements.
